# Epidemiologic Investigations of Bioterrorism-Related Anthrax, New Jersey, 2001

**DOI:** 10.3201/eid0810.020329

**Published:** 2002-10

**Authors:** Carolyn M. Greene, Jennita Reefhuis, Christina Tan, Anthony E. Fiore, Susan Goldstein, Michael J. Beach, Stephen C. Redd, David Valiante, Gregory Burr, James Buehler, Robert W. Pinner, Eddy Bresnitz, Beth P. Bell, the CDC New Jersey Anthrax Investigation Team

**Affiliations:** * Centers for Disease Control and Prevention, Atlanta, Georgia, USA

**Keywords:** *Bacillus anthracis*, anthrax, bioterrorism

## Abstract

At least four *Bacillus anthracis–*containing envelopes destined for New York City and Washington, D.C., were processed at the Trenton Processing and Distribution Center (PDC) on September 18 and October 9, 2001. When cutaneous anthrax was confirmed in a Trenton postal worker, the PDC was closed. Four cutaneous and two inhalational anthrax cases were identified. Five patients were hospitalized; none died. Four were PDC employees; the others handled or received mail processed there. Onset dates occurred in two clusters following envelope processing at the PDC. The attack rate among the 170 employees present when the *B. anthracis–*containing letters were sorted on October 9 was 1.2%. Of 137 PDC environmental samples, 57 (42%) were positive. Five (10%) of 50 local post offices each yielded one positive sample. Cutaneous or inhalational anthrax developed in four postal employees at a facility where *B. anthracis*–containing letters were processed. Cross-contaminated mail or equipment was the likely source of infection in two other case-patients with cutaneous anthrax.

 On October 4, 2001, inhalational anthrax was diagnosed in a Florida man who had no known exposure risk factors (1). The following week, cases of cutaneous anthrax in persons exposed to letters containing a suspicious powder were reported in New York City. The initial investigation showed that four envelopes containing *Bacillus anthracis* spores were mailed through the U. S. Postal Service (USPS) to media outlets in New York City and senate offices in Washington, D.C., in September and October 2001. These four recovered envelopes were postmarked at the USPS Trenton Processing and Distribution Center (Trenton PDC) in New Jersey.

On October 18, cutaneous anthrax was confirmed in a New Jersey postal worker. This prompted the closure of the Trenton PDC and initiation of an investigation in New Jersey. The objectives of the investigation were to determine the extent of the anthrax outbreak in New Jersey, assess potential sources of *B. anthracis* exposure, and prevent additional cases by developing and implementing control measures.

## Methods

### Case Definitions

In this multistate outbreak, all sites adopted the Centers for Disease Control and Prevention (CDC) case definitions for anthrax (2). A confirmed case was defined as a clinically compatible illness that was laboratory confirmed either by isolation of *B. anthracis* from an affected tissue or site, or by two supportive laboratory tests. A suspected case was defined as a clinically compatible illness with no isolation of *B. anthracis* and no alternative diagnosis, but with one positive supportive laboratory test or a clinically compatible illness epidemiologically linked to a confirmed environmental exposure to *B. anthracis*.

Supportive laboratory tests included demonstration of *B. anthracis* in a clinical specimen by immunohistochemical staining; detection of *B. anthracis* DNA by polymerase chain reaction from specimens collected from an affected tissue or site; or the presence of anti-protective antigen immunoglobulin G (anti-PA IgG) by enzyme-linked immunoadsorbent assay (3).

### Case Investigations

Suspected and confirmed case-patients were interviewed about symptoms, employment, and other possible exposures, and their medical records were reviewed. Coworkers and supervisors were also interviewed. For case-patients who were USPS employees, job assignments and time sheets were reviewed, with special attention to dates when letters containing anthrax spores were postmarked. Blood, tissue, and microbiologic samples were obtained and sent for testing. When possible, the incubation period was defined as the time between the date of likely exposure to spore-containing envelopes and the onset of symptoms.

### Surveillance

Initial case finding involved investigation of potential cases reported by health-care providers, hospitals, and the public directly to the health department. Subsequently, we initiated stimulated passive hospital-based surveillance to identify additional inhalational anthrax cases (4). Infection control professionals from 61 hospitals in 15 counties in New Jersey, Pennsylvania, and Delaware, serving an area of 6.7 million residents, provided daily totals of emergency department and intensive-care unit admissions and reported all patients meeting broad clinical criteria (such as respiratory failure or febrile illness without a confirmed alternative diagnosis) for possible inhalational anthrax. Passive surveillance for both inhalational and cutaneous anthrax cases was conducted statewide in New Jersey and in parts of Pennsylvania and Delaware that are contiguous to New Jersey. Surveillance was enhanced through electronic communication with local health departments, press releases, and postings on websites of the New Jersey Department of Health and Senior Services (NJDHSS) and two New Jersey medical associations. All persons with possible anthrax identified through surveillance were followed up through telephone calls to the patients, the physicians and nurses treating them, and requests for laboratory specimens*.*

### Exposure Assessment

To identify locations where exposures to letters containing *B. anthracis* spores might have occurred, we tracked the path of the contaminated letters through the Trenton PDC by obtaining information collected by the USPS for routine tracking and quality control. We also determined how mail flows to and from the PDC as it is brought from and delivered to other postal facilities and to the public.

### Attack Rates

We reviewed the time sheets and specific work locations of the PDC employees working on the night of October 9, when the letters destined for Washington, D.C., were sorted. The number of employees working on this shift and the number of employees working on subsequent shifts were determined by review of available records and interviews with the PDC postmaster. Some records remained unavailable for review because the PDC was closed. We calculated attack rates for inhalational anthrax by dividing the number of cases by the total number of employees in the specified area.

### Environmental Sampling

Initial sampling, conducted October 18–19, focused on the identified path of the letters in the Trenton PDC and public access areas of the PDC. When samples taken from areas along the path of the letters were found to be positive for *B. anthracis* on the following day, we developed a sampling strategy to evaluate the extent of contamination in the building and further characterize the risk to postal employees and visitors.

During October 21–November 9, sampling was conducted in a wider horizontal distribution around the areas of the initial positive samples and vertically upward toward the ceiling of the PDC. Sampling was performed on machinery located beyond the original path of the letters, the ventilation system, lookout galleries (enclosed elevated corridors), administrative areas on the mezzanine level, and the roof rafters. Sampling techniques included swab sampling with sterile moist swabs to collect settled dust and vacuum sock sampling with portable HEPA-filtered vacuum to collect surface dust over large areas (5).

Other mail facilities in New Jersey through which the recognized contaminated letters could have passed were identified and sampled. Most samples from these facilities were collected from areas where the initial mail-sorting activities were conducted. Additional samples were collected from customer areas, receiving bins of indoor mailboxes, cleaning equipment, loading docks, ventilation systems, computer work stations, and at least one delivery vehicle from each site. After the identification of cutaneous anthrax in an office worker who was not a PDC employee, sampling was performed at this case-patient’s workplace and home; the focus was on areas where mail might have been placed or opened.

### Laboratory

*B. anthracis* screening identification of human and environmental samples was performed at the NJDHSS Public Health and Environmental Laboratories according to Bioterrorism Laboratory Response Network Level A and B protocols, with modifications to enhance the recovery rate of spores from environmental samples (6,7). Agar slants with isolates identified as *B. anthracis* by the Public Health and Environmental laboratories were sent to CDC’s Anthrax Laboratory, where identification of *B. anthracis* was confirmed by standard microbiologic procedures and the Laboratory Response Network testing algorithm (6–8). Antimicrobial susceptibility patterns were determined for *B. anthracis* isolates by using National Committee for Clinical Laboratory Standards breakpoints for staphylococci (9). Isolates of *B. anthracis* recovered from clinical specimens and environmental samples were typed to determine their genetic relatedness by using multiple-locus variable-number tandem repeat analysis (MLVA) (10).

### Intervention

Postexposure prophylaxis was made available to potentially exposed persons pending results of environmental testing. We recommended continuation of postexposure prophylaxis for a total of 60 days for persons considered to be at risk for inhalational anthrax (11). A series of three postexposure prophylaxis clinic periods, each involving several sessions, were organized at two local hospitals. Seven or 10 days of antibiotics were dispensed at the initial clinic, and 25 days of antibiotics were dispensed at each of the two follow-up clinics. Hospital staff were available for consultation with persons who could not attend the formal clinics. At the initial clinic, ciprofloxacin was provided. The recommended antibiotic for postexposure prophylaxis was changed to doxycycline for the two follow-up clinics, after testing showed the *B. anthracis* isolates were susceptible to doxycycline (12). Antibiotics were obtained from the National Pharmaceutical Stockpile.

Employees who did not attend the clinics were contacted by telephone and encouraged to come to the clinic. To promote adherence, fact sheets and a newsletter were developed and distributed, reminders for postexposure prophylaxis clinics were posted at work sites, and weekly meetings were held with USPS management and representatives from each of the four postal unions. A health education team conducted focus groups with postal employees and conducted a health education campaign.

## Results

### Demographic and Clinical Characteristics of Cases

From October 18 to October 24, six persons with anthrax were identified in the New Jersey area, including three with confirmed cutaneous anthrax, one with suspected cutaneous anthrax, and two with confirmed inhalational anthrax (Table 1). Their median age was 44 years (range 35–56 years); four were women. Five were USPS employees; four worked at the Trenton PDC, and one was a mail carrier at the West Trenton post office. The sixth case-patient was a bookkeeper at a Hamilton Township, New Jersey, office.

**Table 1:** Characteristics of New Jersey case-patients in the bioterrorism-related anthrax outbreak, September–October 2002

Characteristic	Case 1	Case 2	Case 3	Case 4	Case 5	Case 6
Sex	Female	Male	Male	Female	Female	Female
Age (yrs)	45	39	35	56	43	51
Cutaneous/ inhalational	Cutaneous	Cutaneous	Cutaneous	Inhalational	Inhalational	Cutaneous
Postal worker	Yes	Yes	Yes	Yes	Yes	No
Employed at Trenton PDC^a^	No	Yes	Yes	Yes	Yes	No
Date of illness onset	9/28	9/26	10/14	10/14	10/15	10/17
Incubation period	9 days^b^	8 days	5 days	5 days	6 days	Unknown
Hospitalized	Yes	No	Yes	Yes	Yes	Yes
Survived	Yes	Yes	Yes	Yes	Yes	Yes

^a^PDC, postal distribution center. ^b^Assuming exposure on 9/19.

**Figure 1:**
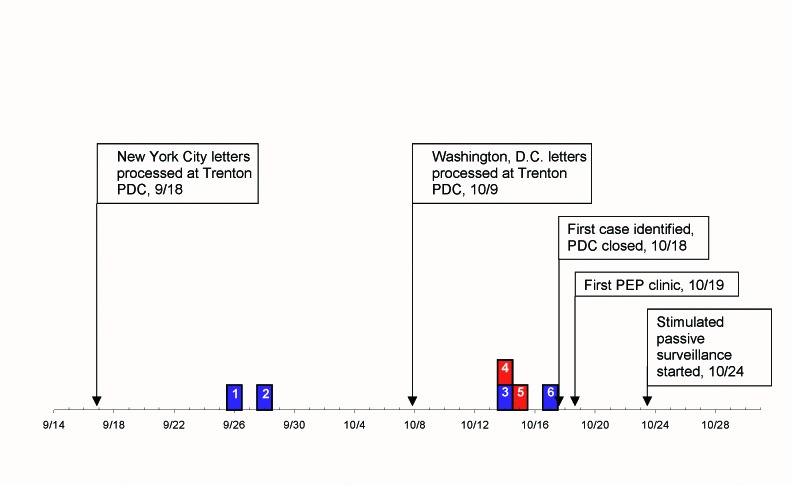
Timeline of events during bioterrorism-related epidemic, New Jersey, September–October, 2001. Red box = l case-patient with onset of inhalational anthrax; blue box = l case-patient with onset of cutaneous anthrax.

The incubation period was 5–9 days (median 8 days) for the three cutaneous cases whose exposure date could be estimated, and 5 and 6 days for the two inhalational cases. The dates of onset were clustered: two case-patients had onset of symptoms 8 and 9 days after the letters sent to New York City were processed at the Trenton PDC on September 18, and four case-patients had onset of symptoms 5–6 days after the letters sent to Washington, D.C., were processed on October 9 (Figure 1). Five of the patients were hospitalized—both persons with inhalational anthrax and three persons with cutaneous anthrax. No case-patients died. Demographic and clinical descriptions of the New Jersey case-patients are summarized in Tables 1–3 and presented in detail elsewhere (1,12–14).

**Table 2:** Initial clinical findings in four patients with bioterrorism-related cutaneous anthrax, New Jersey, September-October 2001^a^

Clinical finding	No. of cases with clinical finding
Physical findings	
Edema surrounding skin lesion	4/4
Black eschar	2/4
Lesion associated with pustules or vesicles	2/4
Tender	2/4
Pruritic	1/4
Laboratory results	
Blood culture positive for *Bacillus anthracis*	1/4^b^
Blood or tissue positive for *B. anthracis* by PCR	2/4
IHC staining positive for *B. anthracis*	3/4^c^
Convalescent-phase serum^d^: anti-PA IgG antibodies present (“reactive serology”)	4/4
Initial diagnosis	
Cellulitis	3/4
Insect bite	1/4


^a^IHC, immunohistochemical staining; PCR, polymerase chain reaction; anti-PA IgG, anti-protective antigen immunoglobulin G. ^b^Only 1/4 patients with cutaneous anthrax had blood cultures drawn before the initiation of antibiotic therapy. This was the one patient with a blood culture positive for *B. anthracis.* ^c^The 4th patient did not have tissue available for IHC staining. ^d^Convalescent-phase serum is serum drawn at least 14 days after symptoms begin.

**Table 3:** Clinical findings in two patients with bioterrorism-related inhalational anthrax, New Jersey, September–October 2001^a^

Clinical finding	Case 1	Case 2
Past medical history	Transient ischemic attack	None
Smoking status	Nonsmoker	Nonsmoker
Initial symptoms	Fever, chills, vomiting, diarrhea	Fever, chills, vomiting, dry cough, headache
Signs at ER visit	Fever: temp=38.4°C; Tachycardia: HR=120/min; Hypoxia: arterial paO2=58 (RA)	Fever: temp=38.4°C; Tachycardia: HR=120/min; Hypoxia: SaO2=92% (RA)
Chest x-ray	Infiltrate, pleural effusion	Infiltrate, pleural effusion
Hospital course	Re-accumulating hemorrhagic pleural effusions	Re-accumulating hemorrhagic pleural effusions
Laboratory results		
Blood culture	Negative (before start of antibiotics)	Negative (after 2 days of antibiotics)
Blood positive for *Bacillus anthracis* by PCR	Yes (before start of antibiotics)	No (after 2 days of antibiotics)
IHC staining of pleural fluid Cytology	Positive for *B. anthracis* cell wall Positive for *B. anthracis* capsule	Positive for *B. anthracis* cell wall Positive for *B. anthracis* capsule
Convalescent-phase serum^b^	Anti-PA IgG antibodies present	Anti-PA IgG antibodies present

^a^ER, emergency room; HIC, immunohistochemical; PCR, polymerase chain reaction; Anti-PA IgG, anti-protective antigen immunoglobulin G. ^b^Convalescent-phase serum is serum drawn at least 14 days after symptoms begin.

### Surveillance

Surveillance was initiated on October 24, and from October 24 to December 17, 2001, hospital infection control practitioners reviewed 240,160 emergency department visits and 7,109 intensive-care unit admissions. Four hundred sixty-four patients who met initial criteria for possible inhalational anthrax were reported to the NJDHSS; 214 (46%) required additional follow-up to rule out inhalational anthrax. Ninety-eight patients with suspicious cutaneous lesions were reported; 26 (27%) were assessed further to rule out cutaneous anthrax. Anthrax was ruled out in all patients; no additional cases were identified (4).

### Exposure Assessment and Mail Flow

**Figure 2:**
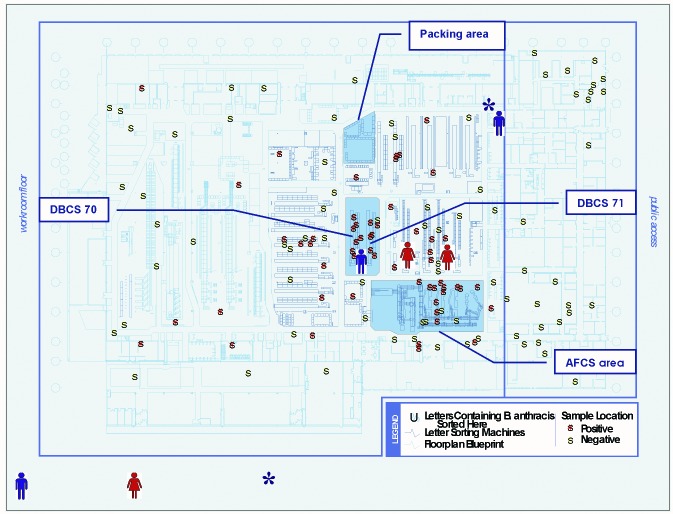
Floor map of the Trenton Postal Distribution Center in Hamilton Township with locations of environmental samples taken October–November, 2001, and work stations of New Jersey case-patients on dates when letters containing *Bacillus anthracis* were sorted. Blue man = male, cutaneous anthrax; red woman = female, inhalational anthrax. *Machine mechanic worked throughout the mail-sorting area the night the letters containing B. anthracis destined for New York were sorted.

The Trenton PDC occupies 281,387 square feet or approximately 7,000,000 cubic feet and is divided into a mail-processing area and administrative and public access areas. Approximately 2 million pieces of mail are processed through the facility each day. The recognized spore-containing letters destined for New York City and Washington, D.C., took similar paths as they were processed through the facility. The letters received a barcode on one of three advanced facer canceller system machines (AFCS) and were then sorted through one of two delivery barcode sorters (DBCS 70 and 71), high-speed machines that read the barcode and sort approximately 30,000 letters per hour into bins according to destination (Figure 2). The letters destined for New York City were sorted through DBCS 70 or 71 in the late afternoon of September 18. Both letters destined for Washington, D.C., were processed in the late afternoon of October 9 through AFCS 3 within approximately 15 minutes of each other, followed by sorting on DBCS 70 within 2 minutes of each other. After sorting, the letters were packed into trays in the packing area and loaded onto mail trucks (Figure 2).

In general, mail that receives the Trenton postmark at the Trenton PDC comes from one of 50 local post offices in central New Jersey, or it is dropped off in a mailbox at the Trenton PDC. We could not determine the source of the letters containing *B. anthracis*. After processing at the Trenton PDC, mail with a Trenton postmark follows one of three routes:1) it is returned to one of the 50 local post offices for local delivery; 2) it is transferred to one of 12 other PDCs in New Jersey, Philadelphia, or Delaware; 3) or it is routed through the transfer facility in Carteret, New Jersey or the airmail center in Newark en route to destinations throughout the world. We confirmed that the recognized spore-containing letters were routed through the Carteret transfer facility en route to their destinations in New York City and Washington, D.C. At the Carteret transfer facility, mail is not unwrapped or handled; it remains in tubs that are transferred from one truck to another.

### Potential Case Exposures

Case-patient 1 (Table 1) was a mail carrier at the West Trenton post office, which sends and receives its mail through the Trenton PDC. She never worked at the Trenton PDC and did not visit that facility. The mail that this carrier delivered on September 19 had been sorted at the PDC on September 18 on the same machines that had sorted the New York City letters earlier that day. On September 28, 10 days later, cutaneous anthrax developed in this mail carrier.

Case-patient 2 (Table 1) was a machine technician. He worked on September 18, 2001, when the letters to the New York City media outlets were sorted. This technician circulated throughout the letter-sorting area, was responsible for maintenance and repair of the high-speed sorters, and used compressed air to blow out dust and debris from the machines. Cutaneous anthrax developed in this man 8 days later.

Case-patient 3 (Table 1) was working on October 9, when the letters containing *B. anthracis* bound for Washington, D.C., were processed. Although he began his shift working in a different area of the facility, he later moved to operate the DBCS 70 that had sorted the letters containing *B. anthracis* earlier that evening (Figure 2). Cutaneous anthrax developed 5 days later.

The two New Jersey inhalational case-patients (Case-patients 4 and 5, Table 1) were also working on the night of October 9. They stood side by side at the input subsystem sorters, machines located next to the AFCS and the DBCS that sorted the *B. anthracis*–containing letters (Figure 2). Inhalational anthrax developed in these case-patients 5 and 6 days later, respectively.

Case-patient 6 was a bookkeeper at a Hamilton Township accounting firm; she did not visit the Trenton PDC. However, mail delivered to both her home and workplace came directly from the Trenton PDC without passing through an intermediate local post office. The bookkeeper’s onset of cutaneous anthrax was October 17, eight days after the Washington, D.C., destined letters were processed at the Trenton PDC.

### Attack Rates for Inhalational Anthrax after Exposure to Washington, D.C.–Destined Letters

The two case-patients with inhalational anthrax (Case-patients 3, 4, Table 1) were identified among 750 Trenton PDC employees who worked in the processing area of the facility during or after the letters addressed to Washington, D.C., were processed on October 9 (overall attack rate 0.25%). The two persons with inhalation anthrax were among 170 who worked in the sorting area on the October 9 shift when the letters transited, for an attack rate of 1.2%.

### Environmental Sampling

Of the 137 samples obtained at the Trenton PDC, 57 (42%) were positive for *B. anthracis* (Figure 2, Table 4). Positive samples were located throughout the facility, including samples taken from rafters as high as 25 feet above the plant floor and samples from the ventilation system (Figure 2). Twenty-five (83%) of 30 samples were positive in the area where the letters containing *B. anthracis* were sorted. Positive samples were identified from the machines at which Case-patients 4 and 5 worked and from the sorting machine that processed mail destined for the workplace and home of Case-patient 6.

**Table 4:** Environmental sampling results of bioterrorism-related anthrax epidemic, New Jersey, October–November 2001

Site	No. of samples	Results
Trenton Postal Distribution Center		
Entire facility	137	57 (42%) positive
Letter-sorting area	30	25 (83%) positive
Customer service area (public area)	20	0 positive
Carteret Transfer Facility	14	0 positive
West Trenton Post Office	57	0 positive
Other 49 local post offices	983	5 (0.5%) positive^a^
		1/72 positive post office #1
		1/19 positive post office #2
		1/15 positive post office #3
		1/18 positive post office #4
		1/24 positive post office #5
Bookkeeper’s home	5	0 positive
Bookkeeper’s workplace	21	1 (4.7%) positive

^a^One each at five distinct facilities.

In addition to the samples collected at West Trenton Post Office, we obtained a mean of 18 samples (range 4–27 samples) from each of 49 local post offices. Five of the local post offices had one positive sample each. The positive sample in each facility came from an area where mail from the Trenton PDC is deposited. One of the samples was obtained underneath a sorting machine, three were obtained from mail containers or the place where mail containers are stored, and one was from a bin inside a mailbox outside the post office. All 54 samples collected from the West Trenton post office (where Case-patient 1 worked) were negative. All 14 samples from the Carteret facility were negative.

Of 21 samples collected from the workplace of Case-patient 6, one grew *B. anthracis*. This sample was obtained from a tray near the receptionist’s desk that held delivered and outgoing mail. None of the samples collected from the home of Case-patient 6 were positive, including samples collected from her mailbox and areas where she stored and opened her mail.

Of the 10 environmental isolates typed by MLVA (4 from locations throughout the Trenton PDC, 5 from the local post offices, and 1 from the workplace of Case-patient 6), all were indistinguishable from clinical isolates.

### Interventions

We recommended 60 days of postexposure prophylaxis for 1,069 employees of the Trenton PDC, as well as for persons who visited the facility and spent >1 hour on the plant floor from September 18 (the date the first letter containing *B. anthracis* was processed in the Trenton PDC) to October 18, 2001 (the date the facility was closed). Beginning October 20, a total of 885 (83%) Trenton PDC postal workers were provided with the full 60-day course of postexposure prophylaxis. Of the 184 (17%) postal workers who did not receive 60 days of antibiotics, 29 (3%) did not receive any antibiotics, 40 (4%) came to the initial clinic only; and 115 (11%) came to the initial and first follow-up clinics. Most postal workers (1,032 [97%]) obtained their antibiotics from Hospital A; 37 (3%) obtained antibiotics from their private physicians.

Three hundred twenty-four visitors to Trenton PDC went to Hospital A (n= 175), Hospital B (n=129), or their private physicians (n=20) for prophylaxis. Of these, 206 (64%) received 60 days of antibiotics, 85 (26%) received <60 days, and 33 (10%) did not receive any antibiotics.

## Discussion

 In New Jersey, *B. anthracis* spores contained in envelopes processed on high-speed mail sorting machines were the source of two cases of inhalational anthrax, four cases of cutaneous anthrax, widespread contamination of the Trenton PDC, and cross-contamination of other letters, equipment, and facilities. Several aspects of the New Jersey outbreak provide insights into how these *B. anthracis* spores were distributed in the environment, the clinical signs and symptoms they caused, and the challenges to public health that arose in the setting of intentional *B. anthracis* contamination.

Envelopes containing *B. anthracis* were handled at the Trenton PDC in a limited area of the facility: they passed through a small number of the many machines used to handle letters. Yet environmental sampling found evidence of spores throughout the facility, including on nearly all of the sorting machines, in the ventilation system, and in the rafters high above the plant floor. These findings are consistent with recent experiments indicating that spores deposited on high-speed sorting machines from the passage of *B. anthracis–*containing envelopes can be readily aerosolized or dispersed through the air and are capable of being carried for considerable distances (15).

Despite evidence of distribution of spores throughout the facility, the epidemiologic investigation demonstrated limited disease. The attack rate among Trenton PDC workers for inhalational anthrax was low, despite the potential for ongoing exposure during the 9 days between the afternoon the letters bound for Washington, D.C., were processed and the day the facility was closed. The two workers in whom inhalational anthrax developed stood next to one another when the letters containing *B. anthracis* were sorted: they worked on machines next to the sorters that processed these letters. Symptoms developed in these workers within 1 day of each other. These findings are consistent with an exposure to a local plume of aerosolized spores during or soon after the passage of the letters. Such a plume could have been produced by air circulation patterns in the vicinity or when compressed air was used to blow out or clean a nearby machine that had processed the letters. We had no means of assessing individual exposure to explore this hypothesis further. For example, all the >900 nasal swabs collected from Trenton PDC workers were negative for *B. anthracis* but were collected at least 10 days after the last known letters were sorted in the facility, perhaps too long after potential exposure to be useful indicators.

The Trenton PDC is the only facility identified in which exposure to letters bound both for New York City and Washington, D.C., occurred, allowing for comparison between the outcomes of these exposures. In New Jersey, only cutaneous anthrax occurred after the letters to New York City were sorted. Although we cannot exclude the possibility that the cases that occurred in temporal association with processing of the Washington, D.C.–destined letters might have been acquired from exposure to the New York City-destined letters, both inhalational and cutaneous anthrax most likely occurred in New Jersey after exposure to the letters to Washington, D.C. Although only inhalational cases were reported in Washington, D.C., these findings are consistent with the predominant forms of anthrax that occurred following exposures to these letters in New York City and Washington, D.C. (12,14). Many factors, including differences in powder or other characteristics in the contaminated letters, as well as differences in environmental or other conditions at the various sites, might account for differences in disease associated with the exposures to the New York City– and Washington, D.C.–destined letters. Ongoing studies of spore and envelope characteristics and aerosol formation during routine mail processing activities might provide further insight.

Two of the six New Jersey cases occurred in persons who did not work at the Trenton PDC and would not have had a direct exposure to a recognized spore-containing letter at any point in the known letter path. In both circumstances, we demonstrated the opportunity for exposure to mail that could have been cross-contaminated when spores deposited in sorting machines or on other equipment were transferred to envelopes subsequently processed in the facility. Although these cases could possibly have resulted from unrecognized direct exposure to envelopes containing *B. anthracis*, we consider exposure to cross-contaminated envelopes to be the probable source of these two cases. Consideration of the potential number of envelopes that might have been cross-contaminated in this fashion gives an appreciation of the rarity of disease from exposure to cross-contaminated envelopes. During the 9-day period after processing of the letters bound for Washington, D.C., before the facility was closed, at least 2 million letters could have been sorted through the same machine that sorted the spore-containing envelopes, and an estimated 18 million pieces of mail would have been processed through the facility. Yet only one case of cutaneous anthrax occurred among the many thousands of USPS employees who handled mail that had passed through the Trenton PDC, and only one case was identified among the many millions of recipients of such envelopes living in our surveillance area. Thus, the risk of anthrax from cross-contamination, while not absent, appears to be quite low.

Given the urgent public health actions that followed the identification of each new case—from facility closures to recommendations for postexposure prophylaxis for hundreds—surveillance played a crucial role in this investigation. We continued surveillance for 8 weeks after the last case had been identified because the outer limit of the incubation period was poorly defined, the extent that mail and other postal facilities had been cross-contaminated was unknown, and there was a possibility that additional *B. anthracis–*containing letters would be posted or other terrorist events would occur. Thus, surveillance was pivotal in demonstrating that the scope of the outbreak was limited to the original cases identified and that the risk to the general population was low. Surveillance also provided a level of assurance that other attacks were not occurring in the area and confirmed that additional public health control measures were not needed. Surveillance also enabled NJDHSS and CDC officials to maintain timely and frequent communication with the health-care community, defined a clear role for health-care providers and hospitals in the response efforts, and provided assurance and consultation to the health-care community and the public.

Effective and frequent communication among postal workers, hospital health-care workers, and NJDHSS and CDC staff members also contributed to the high rate of initiation and completion of postexposure prophylaxis in New Jersey. Some studies have indicated that creating realistic patient expectations about side effects and enhancing patient understanding of illness and treatment promote adherence (16,17). The three postexposure prophylaxis clinics held in New Jersey enabled postal workers to ask questions about anthrax, antibiotic regimens, adverse effects associated with taking the antibiotics, and ways to make taking prophylaxis more tolerable. Close patient follow-up also promotes adherence (16,17), especially when the course of treatment is long. In New Jersey, we made telephone calls to postal workers who did not attend a clinic, and hospital staff were available to see these workers for medication refills outside the formal clinics.

 The New Jersey investigation highlighted unprecedented and unanticipated challenges to public health posed by the intentional release of a pathogenic biologic agent. An urgent public health response led to the rapid development of diagnostic and environmental sampling methods that were refined as the investigation progressed. The implementation of postexposure prophylaxis measures required the development of a large-scale medication delivery infrastructure. Health communication messages were revised daily and often required communicating the uncertainty of risk through the lay media. The possibility of further attacks with anthrax spores or other agents of terrorism remains. Continued vigilance and close cooperation among the various health, law enforcement, and other groups and agencies, as well as continued support of efforts to rebuild and update the public health infrastructure, are needed to protect the public’s health. This relatively limited bioterrorism attack required considerable resources and time from public health, health-care providers and hospitals, and law enforcement. Further evaluation of the New Jersey and other anthrax bioterrorism investigations may prove helpful in developing responses to future attacks.
